# Whole Genome Sequencing-Based Mapping and Candidate Identification of Mutations from Fixed Zebrafish Tissue

**DOI:** 10.1534/g3.117.300212

**Published:** 2017-08-29

**Authors:** Nicholas E. Sanchez, Breanne L. Harty, Thomas O’Reilly-Pol, Sarah D. Ackerman, Amy L. Herbert, Melanie Holmgren, Stephen L. Johnson, Ryan S. Gray, Kelly R. Monk

**Affiliations:** * Department of Developmental Biology, Washington University School of Medicine, St. Louis, Missouri 63110; † Department of Genetics, Washington University School of Medicine, St. Louis, Missouri 63110

**Keywords:** zebrafish, whole genome sequencing, mapping, linkage, genetic screen, fixed tissue sequencing

## Abstract

As forward genetic screens in zebrafish become more common, the number of mutants that cannot be identified by gross morphology or through transgenic approaches, such as many nervous system defects, has also increased. Screening for these difficult-to-visualize phenotypes demands techniques such as whole-mount *in situ* hybridization (WISH) or antibody staining, which require tissue fixation. To date, fixed tissue has not been amenable for generating libraries for whole genome sequencing (WGS). Here, we describe a method for using genomic DNA from fixed tissue and a bioinformatics suite for WGS-based mapping of zebrafish mutants. We tested our protocol using two known zebrafish mutant alleles, *gpr126^st49^* and *egr2b^fh227^*, both of which cause myelin defects. As further proof of concept we mapped a novel mutation, *stl64*, identified in a zebrafish WISH screen for myelination defects. We linked *stl64* to chromosome 1 and identified a candidate nonsense mutation in the *F-box and WD repeat domain containing 7* (*fbxw7*) gene. Importantly, *stl64* mutants phenocopy previously described *fbxw7^vu56^* mutants, and knockdown of *fbxw7* in wild-type animals produced similar defects, demonstrating that *stl64* disrupts *fbxw7*. Together, these data show that our mapping protocol can map and identify causative lesions in mutant screens that require tissue fixation for phenotypic analysis.

Zebrafish (*Danio rerio*) have emerged as an ideal model organism for both genetic and chemical screens due to their vertebrate anatomy and physiology, large clutch sizes, fully sequenced genome, transparency during development, and ease of maintenance. Forward genetic screens in zebrafish have uncovered many new mutant alleles that disrupt key regulators of a wide variety of developmental and cellular biological processes (Driever *et al.* 1996; Haffter *et al.* 1996). However, the rate at which new mutants are generated has not been matched by the rate of linking a mutant phenotype to a specific causative lesion, which has created a backlog of unmapped mutants. Traditional PCR-based mapping methods are extremely time consuming, taking months or even years (Talbot and Schier 1998; Geisler *et al.* 2007; Zhou and Zon 2011). With the relatively recent advent of next-generation sequencing approaches such as whole genome sequencing (WGS) and RNA sequencing, a phenotype can be linked with a genomic region or a specific mutation in a matter of days or weeks (Bowen *et al.* 2012; Leshchiner *et al.* 2012; Obholzer *et al.* 2012; Voz *et al.* 2012; Miller *et al.* 2013).

However, while mapping with WGS can allow rapid identification of causative mutations, this technology has to date only been applied to screens wherein phenotypes can be easily identified by gross anatomical observation or by fluorescent transgenes, which allow genomic DNA (gDNA) extraction from fresh tissue. To our knowledge, WGS-based mapping protocols have not been applied to screening strategies that require tissue fixation such as whole-mount *in situ* hybridization (WISH) or antibody staining. In these strategies, the phenotypes can only be identified after the samples have been fixed and have undergone a variety of other chemical manipulations, which compromise not only the ability to extract sufficient amounts of gDNA, but also the quality of gDNA obtained.

One good example of a biological process that is difficult to screen without tissue fixation is myelination. Myelin is a multilamellar, lipid-rich membrane that is iteratively wrapped around neuronal axon segments. It is produced by oligodendrocytes in the central nervous system (CNS) and Schwann cells in the peripheral nervous system (PNS). Myelin is a jawed vertebrate innovation (Zalc *et al.* 2008) and, as such, zebrafish are the most tractable model organism to screen for genes involved in the formation of myelin and the development of the myelinating glia (Kazakova *et al.* 2006; Pogoda *et al.* 2006). However, while disruptions to myelinating glia result in debilitating symptoms in a wide variety of neurological disorders in humans, disruptions to genes specifically affecting myelination do not typically result in gross morphological abnormalities during early zebrafish development. Further, although there are transgenic lines that label myelinating glia, changes in myelination can be difficult to assess by simple transgenic screening, especially in the PNS. Therefore, one common approach to screen for myelination defects in both the CNS and PNS has been to assess the expression of *myelin basic protein* (*mbp*) by WISH or antibody staining (Kazakova *et al.* 2006; Pogoda *et al.* 2006).

Here we describe methods for extracting gDNA from zebrafish larvae after WISH and present a WGS and bulked segregate analysis (BSA)-based approach to link mutant phenotypes identified in forward genetic screens to a genomic region, and to identify possible causative mutations. We validated our approach using known mutations and report a novel mutation uncovered in a forward genetic screen for regulators of myelination.

## Materials and Methods

### Zebrafish husbandry and genotyping

All *D. rerio* stocks were maintained in the Washington University Zebrafish Consortium facility (http://zebrafish.wustl.edu/husbandry.htm). Embryos were collected from paired matings and raised in egg water (5 mM NaCl, 0.17 mM KCl, 0.33 mM CaCl_2_, 0.33 mM MgSO_4_) at 28.5°. At 5 d postfertilization (dpf), larvae were transitioned to a rotifer-based diet for 10–14 d before incorporating flake food and flowing water. Traditional morphological markers were used to stage embryos (Kimmel *et al.* 1995). When necessary for WISH, development of pigment was prevented by adding 0.003% phenylthiourea (PTU) to egg water ∼24 hr postfertilization (hpf) and maintained until fixation at 5 dpf. All animal experiments were performed in compliance with Washington University’s institutional animal protocols.

### Forward genetic screen

A standard three-generation forward genetic screen was performed in zebrafish using the chemical mutagen, N-ethyl-N-nitrosourea (ENU) (Mullins *et al.* 1994; Solnica-Krezel *et al.* 1994). Briefly, adult wild-type (WT) (SAT strain) males were mutagenized using 3–3.5 mM ENU over the course of 4–6 wk, allowed to recover for 1 month, and then crossed to WT (SAT) females, resulting in the F1 generation (Draper *et al.* 2004). Each F1 individual was then outcrossed to a double transgenic line [*tg(lhx1a:egfp)* (Swanhart *et al.* 2010); *tg(mbp:mcherry-CAAX)* (kind gift from D. Lyons, University of Edinburgh), on a mixed-strain WT background], which marked a subset of neurons (*lhx1a*) and all myelinating glia (*mbp*). This cross produced F2 families, which were raised together and then intercrossed to drive putative mutations to homozygosity in the F3 progeny. Clutches of F3 larvae that showed altered *mbp* promoter expression by transgene were selected for verification using WISH to directly assess *mpb* expression. When initial transgenic screening proved to be inconsistent, all subsequent screening was done exclusively by WISH using the *mbp* riboprobe.

### WISH

We used standard protocols (Thisse and Thisse 2008) to perform WISH on zebrafish larvae. After 24 hpf, embryos were raised in egg water with 0.003% PTU to prevent the development of pigment. At the desired developmental stages, pools of 25–40 larvae were anesthetized and then fixed in 4% paraformaldehyde (PFA) for 2 hr at room temperature or 4° overnight with gentle agitation. After fixation, samples were then dehydrated in 100% methanol overnight at −20°. Following dehydration, embryos were washed in 0.2% PBS-Tween (PBSTw), permeabilized with proteinase K (20 mg/µl diluted 1:1000 in 0.2% PBSTw), and incubated with digoxigenin-labeled riboprobe overnight at 65° in hybridization buffer (50% formamide). Following probe treatment, embryos were washed and then blocked for at least 1 hr at room temperature in a solution of 2% blocking reagent made in maleic acid buffer with 0.2% triton (MABTr) supplemented with 10% normal sheep serum. Samples were then incubated overnight at 4° in primary antibody [Anti-Dig, Fab fragments (1:2000); Roche, Pleasanton, CA] diluted in blocking solution, with gentle agitation. Following primary antibody treatment, embryos were repeatedly washed in MABTr and developed by alkaline phosphatase reaction. Embryos were then postfixed in 4% PFA, passaged through increasing concentrations of glycerol, and stored long term in 70% glycerol at 4°, protected from light. The *mbp* (Brösamle and Halpern 2002) and *nkx2.2a* (Barth and Wilson 1995) riboprobes have been previously described. All scoring was performed blinded to genotype.

### Genotyping egr2b^fh227^ and fbxw7^stl64^

DNA for genotyping was extracted by adding 50 µl of fish lysis buffer (10 mM pH 8 Tris, 1 mM EDTA, 0.3% tween, and 0.3% glycerol) to tissue. After cooling from a 10-min digestion at 98°, 10 µl of 10 mg/ml proteinase K was added to each sample, and all samples were digested for 12 hr at 55°. The proteinase K was inactivated by incubating at 98° before storing the DNA for later use. This process tended to provide low amounts of fragmented gDNA that was sufficient for genotyping but not sufficient for WGS.

The *egr2b^fh227^* allele was genotyped by using the primers 5′-GAGGACTTTCGCTCTTTTTG-3′ and 5′-TCGGACGAACTTACCAGACAC-3′, which amplified a 228-bp region including the *egr2b^fh227^* mutation. These primers were redesigned from those described in Monk *et al.* (2009) to better amplify the smaller sized fragments of gDNA that were typically extracted from fixed tissue. A 40-cycle PCR with a 60° annealing temperature and 45-sec extension time was performed on all samples. To assay the *egr2b^fh227^* mutation status, disrupts of an *Nsi*I restriction site was used as described previously (Monk *et al.* 2009). PCR amplicons were digested with the *Nsi*I enzyme (New England Biosciences, Ipswich, MA) for 2 hr at 37°, and then heat inactivated for 20 min at 65°. The sizes of digested products were visualized using a 3% agarose gel with ethidium bromide. Samples with two bands at 161 and 67 bp were scored as *egr2b^+/+^* (WT), a single uncut band at 228 bp was scored as *egr2b^fh227/fh227^* (mutant), and individuals with all three size bands were called *egr2b^fh227/+^* (heterozygous).

The *fbxw7^stl64^* mutation was genotyped using the primers 5′-CTCTCCAGTGTGACCAGGTT-3′ and 5′-GCTCTCAGGTCCTCACAAGC-3′, which amplify a 147-bp region surrounding the *stl64* lesion. PCR was performed for 40 cycles with an annealing temperature of 55° and an extension time of 45 sec. The *stl64* mutation disrupts a *Hpy*188I restriction site, so this disruption was used to genotype individual fish. PCR amplicons were digested with the *Hpy*188I enzyme (New England Biosciences) for at least 1–2 hr at 37°, and then heat inactivated at 65° for 20 min. Products sizes were visualized on a 2–2.5% agarose gel with ethidium bromide. A thick band around 75 bp was scored as *fbxw7^+/+^* (WT), a single uncut band at 147 bp was scored as *fbxw7^stl64/stl64^* (mutant), and individuals with both size products were called *fbxw7^stl64/+^* (heterozygous).

### gDNA extractions from fresh tissue

Pools of anesthetized fish in egg water were collected in 1.5-ml tubes, and as much liquid as possible was removed from each tube. 500 µl of fish lysis buffer was added, and the tubes were heated at 98° for 10 min before cooling. Next, 5 µl of 20 mg/ml proteinase k was added, and all tubes were incubated overnight at 55° with gentle agitation. After digestion, the samples were spun in a centrifuge at 17,900 × *g* for 1 min. The resulting supernatant was moved to a new tube and 500 µl of 100% isopropanol was added. The samples and isopropanol were incubated overnight at −20° and then spun for 15 min at 17,900 × *g* at room temperature. After centrifuging, the supernatant was removed and 500 µl of 70% ethanol was added. This mixture was then incubated at room temperature for at least 5 min before as much liquid as possible was removed with a pipette. Finally, the sample was air dried and the resulting pellet was suspended in TE for later use.

### gDNA extractions from fixed tissue

For *egr2b^fh227^* mutants, after *mbp* WISH was completed, zebrafish larvae were passaged into 70% glycerol and scored for *mbp* expression. Mutant animals were separated from their phenotypically WT siblings, and phenotypically WT and mutant larvae were pooled separately. These pools were then backed out of glycerol by replacing the 70% glycerol with 50% glycerol for 5 min, and then replacing the 50% glycerol with 30% glycerol for another 5 min. The 30% glycerol was then replaced with PBSTw. On the other hand, *stl64* mutants were not passaged into glycerol before scoring and pooling, and were instead pooled directly into PBSTw. For both *egr2b^fh227^* and *stl64* mutants, all samples were washed at least three times in PBSTw. Finally, as much PBSTw as possible was removed, and gDNA was extracted using the DNeasy Blood & Tissue Kit (Qiagen cat# 69506) with four key modifications to the manufacturer’s protocol.

These four modifications substantially increased our gDNA yield; specifically, we: (1) incubated for 10 min at 56° when the samples were in the ATL buffer, (2) incubated for a minimum of 3 hr at 200 rpm during the proteinase-k digestion, (3) incubated for 10 min at 56° when the samples were in AL buffer, and (4) incubated at least 20 min (can go longer; *e.g.*, >90 min) after the buffer AE is added before centrifuging for 2 min. Additionally, we found that it is of paramount importance to use a fresh kit, ideally <3 months old, to process samples. Using older kits resulted in significantly decreased or failed extraction.

### WGS

All gDNA was extracted and submitted to the Genome Technology Access Center (GTAC) at Washington University for WGS. Quantification of concentration and integrity of all gDNA was determined by using Qubit (ThermoFisher) and TapeStation (Agilent), respectively. All samples submitted had a minimum of 1 µg of gDNA. Each sample was barcoded, pooled, and paired-end sequencing was done in a single lane of a HiSeq2500 or 3000 (Illumina). After demultiplexing, all reads were aligned using NovoAlign (Novocraft). From the alignments, SNPs and insertions and deletions (INDELS) were called using SAMtools (Li *et al.* 2009; Li 2011), and any effects to protein-coding genes were predicted using snpEFF (Cingolani *et al.* 2012).

### BSA

There are three custom Perl scripts, referred to as “ChromSplit,” “Allele Ratio Calculator (ARC),” and “SNPfilter,” that link mapping and filtering of causal variants. ChromSplit takes the bam file generated in the process of aligning the reads to the genome and splits them into two files per chromosome. The first contains all SNPs called and the second contains all INDELS observed. SNPs and INDELS observed in the mitochondrial genome or on unattached chromosomal scaffolds are discarded prior to linkage mapping. ARC tiles bins of custom size across the different chromosomes and fills each bin with the ratio of mutant to reference alleles for all SNPs observed in both the mutant and sibling pools. When all SNPs are sorted into their appropriate bins, the ratio of mutant to reference alleles is calculated for the whole bin, and that ratio is compared between the mutant and sibling pools. The mutant-to-sibling pool ratio for each bin is generated by ARC.

With the output of ARC, all bins with <10 SNPs are thrown out because of the outsized effect that a single SNP can have. To determine a linked chromosome, the ratios of the mutant and sibling bins are graphed and the highest peak that approaches a ratio of two indicates the linked chromosome. To identify the region of a single chromosome that is most linked to the phenotype used to sort pools, all bins from that chromosome were sorted by their ratios. The 20 bins with the highest ratios are selected, and any area where those selected bins are clustered is the region of the chromosome most closely linked to the phenotype.

### SNP filtering and prioritization

The SNPfilter script takes the output from the snpEFF program and sorts out all SNPs and INDELS previously observed in unmutagenized strains as well as SNPs and INDELS previously observed as homozygous that are not causative. The database of variants from unmutagenized lines used here was the SNPFisher database (Butler *et al.* 2015). SNPfilter was written to use the SNPFisher database as a base while adding the homozygous SNPs observed in the snpEFF files being filtered to the database of known SNPs for future filtering. In this way, as SNPfilter is run on more samples, its ability to filter out noncausative SNPs and INDELS increases. Any SNPs not removed by SNPfilter that are homozygous and nonsynonymous are candidates for the phenotype used to sort the mutant and sibling pools.

### Transmission electron microscopy

Transmission electron microscopy (TEM) was performed between body segments 5–7 of 8.5 dpf zebrafish larvae using established protocols employing microwave assistance (Czopka and Lyons 2011). In brief, individual larval trunks were fixed in modified Karnovsky’s fixative (4% PFA, 2% glutaraldehyde, 0.1 M sodium cacodylate, pH 7.4) via microwave assistance and then overnight at 4°. After genotyping and pooling samples with the same genotypes, samples were washed with 0.1 M sodium cacodylate and postfixed for 1 hr in 2% osmium tetroxide in 0.1 M sodium cacodylate and 0.1 M imidazole. Samples were then washed with ultrapure water and stained with saturated uranyl acetate. Larvae were dehydrated using increasing concentrations of ethanol and then 100% acetone, and finally were infiltrated overnight with a 1:1 acetone:EPON mix at room temperature, using gentle agitation of the tubes. The next day, samples were transferred to 100% EPON, while allowing any residual acetone to fully evaporate. Finally, individual larvae were embedded in 100% EPON and baked for at least 48 hr at 65°.

When the EPON was solid, excess material was trimmed off each block and thin (70 nm) sections were mounted on copper mesh grids (Electron Microscopy Sciences, Hatfield, PA). Grids were stained again with saturated uranyl acetate for 1 hr, rinsed with ultrapure water, and then stained with Sato’s lead stain for 6 min. Grids were imaged using a Jeol (JEM-1400) electron microscope and images were collected using an AMT V601 digital camera. All images were analyzed using the FIJI module of ImageJ and Adobe Photoshop.

### Morpholino injections

A morpholino targeting *fbxw7* has been previously described and validated (Snyder *et al.* 2012). For injections, the morpholino was diluted in ultrapure water supplemented with phenol-red dye (10%) to obtain a final concentration of 2.5 µg/µl. Embryos were injected at or before the one-cell stage with 2.5 ng of morpholino in a total volume of 2 nl. To control for potential adverse effects of the injections, control siblings were also injected with an equal volume of phenol-red dye diluted 1:10 in ultrapure water. Finally, we scored morpholino and control-injected animals by *mbp* WISH. All scoring was performed blind to treatment.

### Data availability

The .bed files produced during sequencing the *stl64* mutation, the three Perl scripts, and a guide to using these scripts are freely available (https://zenodo.org/record/843605).

## Results

The specialized glia that produce myelin provide vital trophic support to ensheathed axons, while the myelin sheath itself protects axons and allows for saltatory conduction of action potentials (Nave 2010). The first forward genetic screens in zebrafish for disruptions in myelinating glial cell development used WISH to assess changes in the levels or patterns of *mbp* expression in the CNS and PNS (Kazakova *et al.* 2006; Pogoda *et al.* 2006). Over the years, as the causative mutations for these mutant phenotypes were identified, the study of these genes has contributed tremendously to the understanding of the development of myelinating glia. However, there are still many aspects of myelinating glial cell development and myelination that remain mysterious.

Therefore, to identify novel regulators of oligodendrocyte and Schwann cell development, we conducted a large-scale, three-generation forward genetic screen in zebrafish. The genomes of 80 adult male zebrafish were randomly mutagenized with ENU to produce the founder generation. We then drove these mutations to homozygosity and assessed the third generation (F3) progeny for changes in *mbp* expression patterns in the CNS and PNS by WISH using a riboprobe for *mbp* (Brösamle and Halpern 2002). The extensive tissue fixation and chemical manipulation of these samples precluded known WGS-based methods for mapping putative causative mutations for any phenotypes recovered. Therefore, to take advantage of the power and speed of WGS in mapping mutations, we developed a WGS–BSA pipeline using gDNA isolated from fixed tissue.

### The WGS–BSA pipeline accurately maps the st49 allele to gpr126 using gDNA from fresh tissue

**Figure 1:**
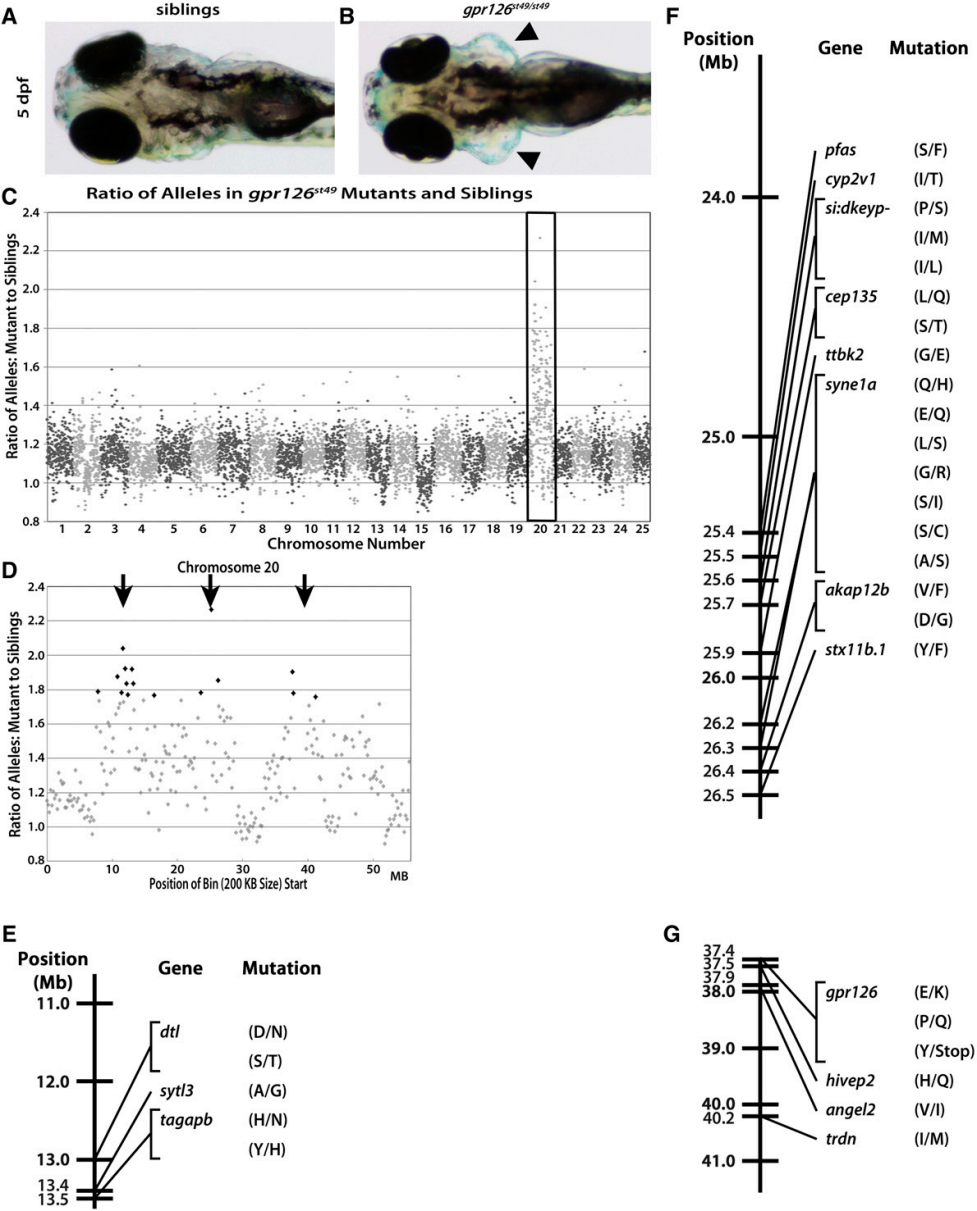
The *st49* allele is accurately mapped to chromosome 20 and *gpr126* using gDNA extracted from fresh tissue. Dorsal views of phenotypically WT (A) and *gpr126^st49/st49^* (B) zebrafish show the characteristically enlarged ears (arrowheads) of mutants at 5 dpf. (C) When the ratio of variant to reference alleles in the mutant pool is compared to the sibling pool and graphed across the whole genome for *gpr126^st49^*, there is a clear spike on chromosome 20 for the *gpr126^st49^* mutation (box). This spike indicates linkage of the region to the trait used to sort the mutant and sibling pools. (D) *gpr126^st49^* is linked to three separate regions on chromosome 20 (arrows). (E–G) Linkage map of the *gpr126^st49^* allele showing the three different regions of chromosome 20 linked to the expanded ear phenotype that was used to sort the mutant and sibling pools. Between all three linked regions there are 29 different protein-coding, homozygous, nonsynonymous SNPs. The single introduced stop is the mutation responsible for the *gpr126^st49^* mutant phenotype.

To establish our pipeline, we first sequenced a known mutation that results in phenotypes easily observed by gross morphology so that we could test the ability of our WGS analysis scheme to correctly identify a causative lesion. The *st49* mutant allele was uncovered in a forward genetic screen in zebrafish for mutants with developmentally disrupted *mbp* expression (Pogoda *et al.* 2006). The causative mutation was later identified through traditional PCR-based linkage mapping and Sanger sequencing of genes in the linked area as a T-to-A transition, resulting in a nonsense mutation in the gene *adgrg6/gpr126* (Monk *et al.* 2009). Importantly, in addition to decreased *mbp* expression in the PNS observed by WISH, at 5 dpf, *gpr126^st49/st49^* animals display a swollen ear phenotype (Figure 1, A and B) that readily distinguishes mutant animals from their WT and heterozygous siblings in living larvae (Monk *et al.* 2009; Geng *et al.* 2013). We took advantage of this obvious morphological defect to collect a pool of phenotypically mutant larvae with swollen ears and a pool of siblings that displayed phenotypically normal ears (*N* = 30 larvae per pool).

We then extracted gDNA from each pool and submitted both samples to the GTAC at Washington University for WGS on a single lane of the Illumina HiSeq3000. Sequencing reads for both pools were aligned to the Zv9 genome build of the zebrafish genome using NovoAlign (Howe *et al.* 2013) (Table 1), and variants (SNPs, insertions, and deletions) were called using SAMtools (Li *et al.* 2009; Li 2011) and annotated using snpEFF (Cingolani *et al.* 2012). Our sequence analysis pipeline consists of three different custom Perl scripts that can be run locally on any workstation and easily tailored to specific circumstances. Henceforth, these scripts will be referred to as “ChromSplit,” “ARC,” and “SNPfilter.”

**Table 1:** WGS coverage of the *gpr126*, *egr2b*, and *stl64* pools

	Total Mapped Reads	Total Aligned Read Pairs	% Genome ≥ 5× (%)	% Genome ≥ 10× (%)
*gpr126^st49^* siblings	63,359,360	27,209,088	67.34	49.39
*gpr126^st49^* mutants	58,637,164	24,936,351	66.59	49.37
*egr2b^fh227^* siblings	95,221,594	44,326,398	74.86	46.83
*egr2b^fh227^* mutants	93,143,924	43,264,449	74.86	47.31
*stl64* siblings	99,225,270	41,977,069	65.45	20.94
*stl64* mutants	116,915,836	49,043,710	69.69	30.12

Coverage statistics for each pool sequenced. Mapped reads are counted as mapped when an individual read is mapped to the genome without regard for its pairs. Aligned pairs are counted as only those reads where both reads of the pair were able to be aligned. All alignment calculations are in regard to the ability of NovoAlign to map a read accurately. Coverage calculations are based off the actual depth of coverage across the Zv9 build of the zebrafish genome.

For both sibling and mutant data sets, we used the ChromSplit script to split each variant in the BED file based on genomic position into separate files for each of the 25 zebrafish chromosomes. Mitochondrial DNA and variants currently assigned to genomic scaffolds were excluded. The second script, ARC, then used the 50 individual chromosome files, 25 sibling and 25 mutant, to group variants into nonoverlapping bins based on chromosomal position and to calculate the mutant allele frequency (MAF) in each bin. As a starting point, we set the bin size to 200 kb. In total, 6735 bins of a 200-kb size were generated, containing at least one SNP with the variant allele being seen in some frequency in both the sibling and mutant pools. Finally, using ARC we compared the MAF for each bin between siblings and mutants to calculate the ratio of homozygosity between these two groups. Any bins with <10 SNPs were subsequently removed due to the outsized ability of any outliers to affect the calculated ratios. In this instance, 6531 bins had 10 or more SNPs with variant alleles seen in both the mutant and sibling *st49* pools data.

To determine the specific chromosome linked to the mutant phenotype, we graphed the mutant:sibling allele ratios for all bins by genomic position and looked for regions of high homozygosity as seen by sequential bins with an allele ratio of ∼2. For a variant that is homozygous in the mutant pool and with perfect Mendelian ratios in the sibling pool, the theoretical expected allele ratio/mapping score is three at single base pair resolution. We expect our mapping scores to approach this limit but to rarely, if ever, reach it because we use a nonoverlapping bin size of 200 kb to calculate the mapping score. The overall allele ratio/mapping score is reduced in the individual bins because we are using a large bin filled, in some cases, with hundreds of SNPs from highly heterogenous genetic backgrounds several generations removed from isogenic mapping strains. Additionally, we do not control for the genotypic ratio in the sibling pool, thus in practice we observe allele ratios closer to two for bins with high rates of homozygosity. When graphing the mutant:sibling allele ratios of all the bins across the entire genome for *st49* mutants, there was a clear peak at chromosome 20, indicating linkage to the swollen ear phenotype (Figure 1C). When viewing only chromosome 20, three distinct regions displayed the highest levels of SNP ratio imbalances between the mutant and sibling pools, indicating linkage of these regions to the mutant phenotype used to sort the pools. These three distinct regions were centered around chromosomal positions 12, 25, and 39 Mb (Figure 1D).

Finally, we used SNPfilter to eliminate variants in protein-coding sequences that had been previously annotated by SNPFisher (Butler *et al.* 2015), a database of SNPs observed in nonmutagenized zebrafish. This process eliminated SNPs that were present in WT populations of zebrafish, and therefore were unlikely to cause our phenotype of interest. All SNPs annotated by snpEFF as protein coding, that resulted in nonsynonymous amino acid changes, and that were verified to be homozygous in the mutants but not siblings using the integrative genomics viewer (IGV) (Robinson *et al.* 2011; Thorvaldsdóttir *et al.* 2013) were considered putative causative lesions. The *st49* mutant pool had 71,791 SNPs annotated by snpEFF (Table 2) as coding SNPs. After filtering out known SNPs, 35,730 SNPs remained. Only 29 SNPs fall into any of the three intervals linked to the *st49* mutant phenotype (Figure 1, E–G). Of all the SNPs that pass all filtering, the SNP predicted to be the most deleterious was a T-to-A nonsense mutation in *gpr126*, which is the causal mutation for the *gpr126^st49^* allele (Monk *et al.* 2009).

**Table 2:** Number of exonic SNPs and INDELS in the *gpr126*, *egr2b*, and *stl64* pools

	Number of Exome SNPS	Number of Exome INDELS
*gpr126^st49^* siblings	74,146	1843
*gpr126^st49^* mutants	66,055	1798
*egr2b^fh227^* siblings	153,942	2862
*egr2b^fh227^* mutants	155,256	2884
*stl64* siblings	126,026	2644
*stl64* mutants	160,112	3275

Number of mutations, by SNPs and INDELS, to the reference genome (Zv9) for all pools sequenced.

### The WGS–BSA pipeline accurately maps the fh227 allele to egr2b using gDNA from fixed tissue

**Figure 2:**
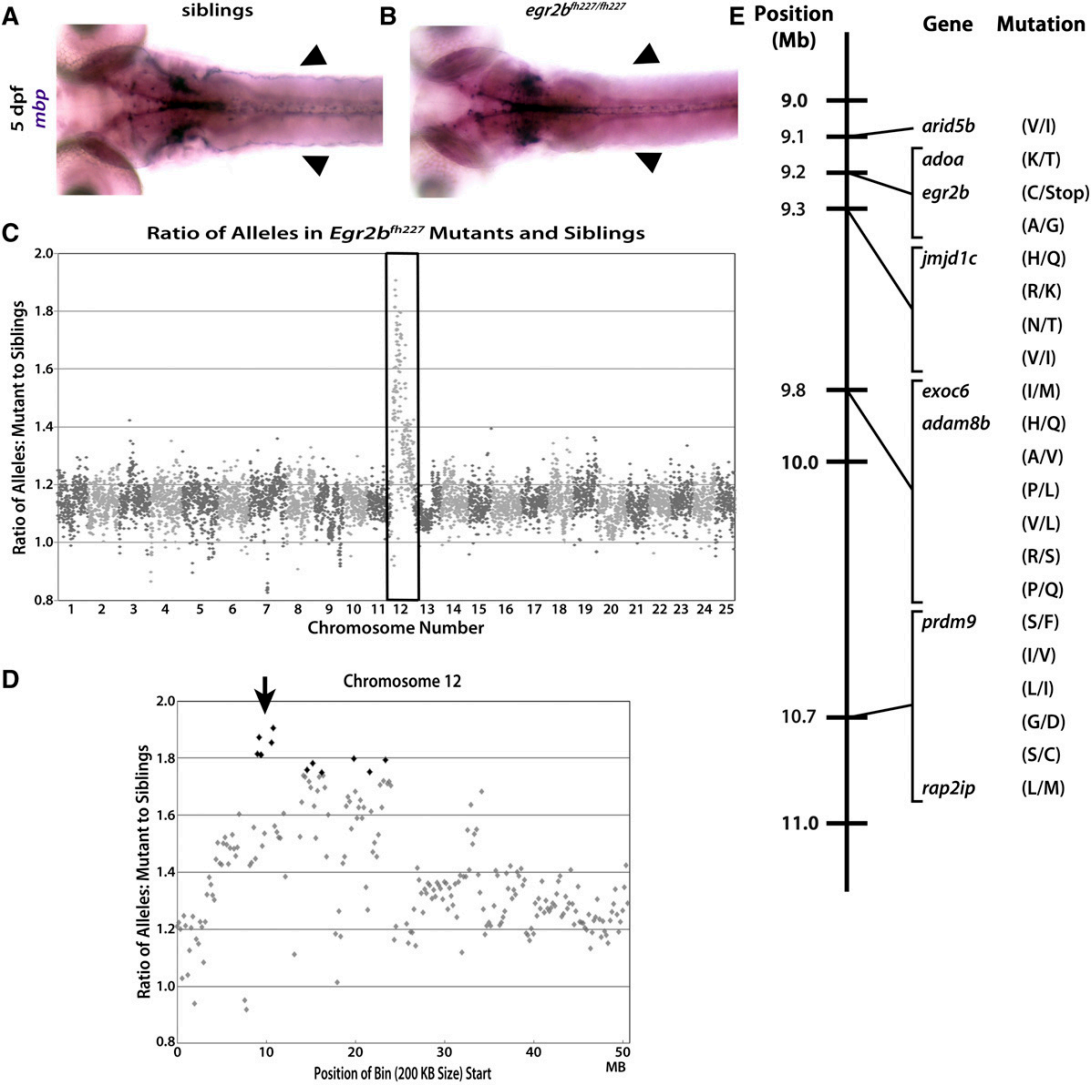
The *fh227* allele is accurately mapped to chromosome 12 and *egr2b* using gDNA extracted from fixed tissue. Dorsal view of *mbp* expression in 5 dpf zebrafish using WISH and the *mbp* riboprobe shows phenotypically normal expression (purple) of *mbp* along the pLLNs (arrowheads) (A) compared to severely reduced *mbp* expression along the pLLN (arrowheads) in *egr2b^fh227/ fh227^* mutants (B). (C) When the ratio of variant to reference alleles in the mutant pool is compared to the sibling pool and graphed across the whole genome for *egr2b^fh227^*, a clear spike on chromosome 12 is observed (box). This spike indicates genotypic linkage to the trait used to sort the mutant and sibling pools. (D) When looking at chromosome 12, *egr2b^fh227^* is linked to a single region centered ∼10 Mb (arrow). (E) Linkage map of the *egr2b^fh227^* allele showing the 21 different homozygous, nonsynonymous, protein-coding SNPs in the single chromosome 12 region linked to the decreased *mbp* expression in the PNS which was used to sort the mutant and sibling pools. The single introduced stop is the mutation responsible for the *egr2b^fh227^* mutant phenotype.

Our WGS analysis pipeline accurately predicted the *st49* allele to be a nonsense mutation in *gpr126*, but the gDNA used for WGS was from fresh tissue. To determine if we could use the same process to map mutants from our WISH-based forward genetic screen, we tested our pipeline using a mutation known to disrupt *mbp* expression in the PNS by WISH. The *fh227* allele was discovered through targeting induced local lesions in genomes (TILLING) (Moens *et al.* 2008) for mutations in *egr2b* (*krox20*) and is a C-to-A point mutation that leads to a premature termination codon (PTC) (Monk *et al.* 2009). *egr2b^fh227^* mutants display severely decreased *mbp* expression in the posterior lateral line nerve (pLLN) of zebrafish (Monk *et al.* 2009). Zebrafish genotyped as *egr2b^fh227/+^* were intercrossed and WISH was performed on the resulting progeny at 5 dpf using an *mbp* riboprobe. Individuals were pooled based on the mbp expression phenotype: either phenotypically WT (Figure 2A; *N* = 33) or reduced *mbp* expression (Figure 2B; *N* = 33).

To extract gDNA from fixed WISH larvae with high yields, we modified the animal tissue (column-based) protocol included with the Qiagen DNeasy Blood & Tissue Kit. Two of these modifications were incubating for 10 min at 56° in ATL buffer and again when samples are in AL buffer. Per the manufacturer, the ATL and AL buffers occasionally generate precipitates that can be prevented by briefly warming at 56°. Therefore, we added short incubations as precautions given the precious nature of the samples. To increase gDNA yield, we made two additional modifications: the proteinase-K digestion was increased from 30 min to 3 hr, and the elution incubation was lengthened to at least 20 min. We found that using the recommended digestion and elution times resulted in at least a 10-fold lower yield. Thus, since many noncolumn-based gDNA extraction protocols for a variety of animal tissues involve proteinase-K digestion steps of 12 hr or more, we increased the digestion time to 3 hr and elution time to >20 min and saw a dramatic improvement in gDNA yield (from ∼2–10 ng/μl to ∼75–150 ng/μl). Digestion times >3 hr did not continue to notably increase yield.

All samples were submitted for WGS using the same specifications as described above to map *st49*. We also subjected the *fh227* WGS data to the same analysis paradigm as outlined for *st49*. In total, 6783 bins of a 200-kb size were generated for the *fh227* pools, 6725 of which contained 10 or more SNPs. We then graphed the mutant:sibling allele ratio by genomic position and found a clear peak of homozygosity on chromosome 12, indicating linkage to the *fh227* phenotype (Figure 2C). When viewing only chromosome 12, the most divergent region between mutant and sibling pools, and thus most linked to the *fh227* phenotype, is a single distinct genomic region centered around the 10-Mb mark (Figure 2D).

The *fh227* mutant pool had 167,060 SNPs annotated by snpEFF as coding SNPs (Table 2). After filtering out known SNPs, 81,832 SNPs remained. Notably, only 21 SNPs fell into the interval linked to the *fh227* mutant phenotype (Figure 2E). Of all SNPs that passed all filtering, the SNP predicted to be the most deleterious was a nonsense mutation in *egr2b*, which is the known causal mutation for the *fh227* allele (Monk *et al.* 2009).

### The WGS–BSA pipeline accurately maps the novel stl64 mutation to fbxw7

**Figure 3:**
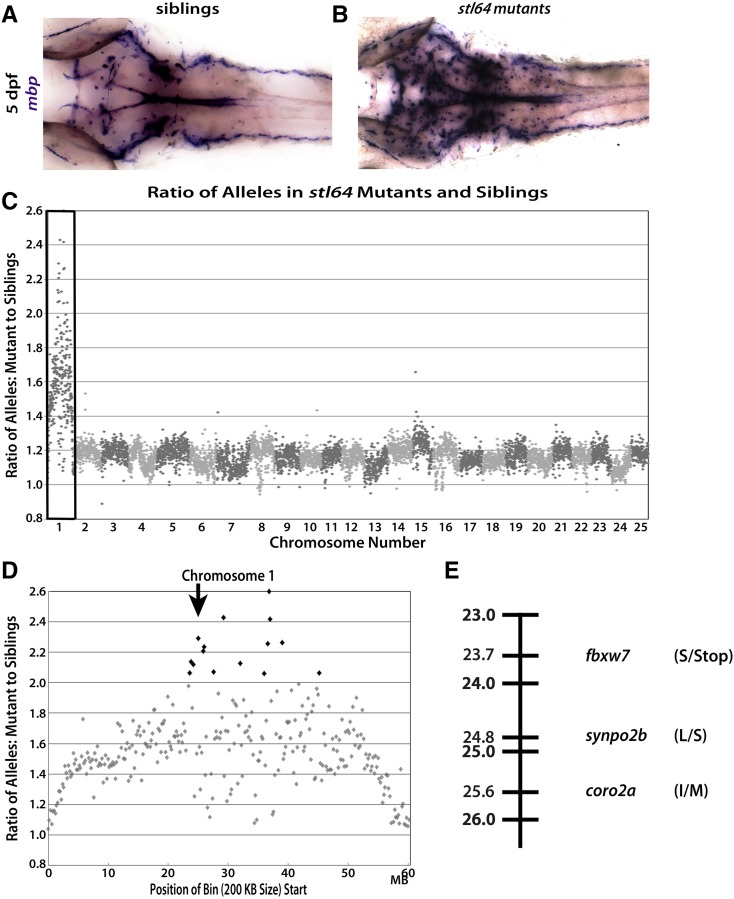
The *stl64* phenotype is linked to chromosome 1 and is likely caused by a nonsense mutation in *fbxw7*. Dorsal views of 5 dpf zebrafish showing *mbp* expression by WISH using the *mbp* riboprobe. Normal expression in the CNS by phenotypically WT siblings (A) compared to the dramatic overexpression of *mbp* in the *stl64* mutants (B) shows increased *mbp* expression in the *stl64* mutants. (C) When the ratio of alleles in the mutant pool compared to the sibling pool is graphed across the whole genome for the *stl64* allele, a clear spike on chromosome 1 (box) is observed for *stl64*. This spike indicates genomic linkage to the trait used to sort the mutant and sibling pools. (D) When viewing chromosome 1, *stl64* is linked to a single region of the chromosome centered ∼24 Mb (arrow). (E) Linkage map of the *stl64* allele showing the three protein-coding, homozygous, nonsynonymous SNP linked to the increased *mbp* expression in the CNS which was used to sort the mutant and sibling pools. The most deleterious SNP is the introduced stop codon in *fbxw7*.

We have demonstrated that we can use gDNA from fixed tissue and successfully perform WGS with that DNA. We have also shown that we can use BSA to link a phenotype to a specific genomic region, and through SNP filtering, we can identify the specific causative mutation. To demonstrate that we can successfully map a novel allele, we ran one of the first mutants from our screen though the WGS–BSA pipeline. This mutant allele, provisionally designated *stl64*, displayed striking overexpression of *mbp* in the CNS when compared to siblings at 5 dpf (Figure 3, A and B).

To determine the causative lesion in the *stl64* mutants, we crossed *stl64* carriers and used WISH with an *mbp* riboprobe to identify mutants. As before, we pooled the larvae into two groups based on their *mbp* expression phenotype: normal *mbp* levels (Figure 3A; *N* = 23) and enhanced *mbp* expression (Figure 3B; *N* = 26). gDNA from both samples was submitted for WGS on the Illumina HiSeq2500 as described above for the *st49* and *fh227* alleles. Sequencing reads for both pools were aligned using NovoAlign to the same Zv9 genome build of the zebrafish genome as was used to map *st49* and *fh227* (Table 1). SNPs were again called and annotated using SAMtools and snpEFF. In total, 6790 bins of a 200-kb size were generated for the *stl64* pools, of which 6746 bins contained 10 or more SNPs. The mutant:sibling allele ratios for *stl64* were highest on chromosome 1 (Figure 3C). Closer inspection of chromosome 1 showed that the ratios of SNPs in the mutant and sibling pools were most different, and thus most highly linked, at a single distinct region centered around chromosomal position 23 Mb (Figure 3D).

The *stl64* mutant pool had ∼155,550 SNPs annotated by snpEFF as coding SNPs (Table 2), 84,553 of which remained after filtering out known SNPs. Of those 84,553 SNPs, only three SNPs fell into the interval linked to the *stl64* mutant phenotype, were protein coding, and were verified in IGV as homozygous in the mutant pool but not in the sibling pool (Figure 3E). Among these three SNPs, the SNP predicted to be the most deleterious was a C-to-A transition resulting in a PTC in *fbxw7*.

**Figure 4:**
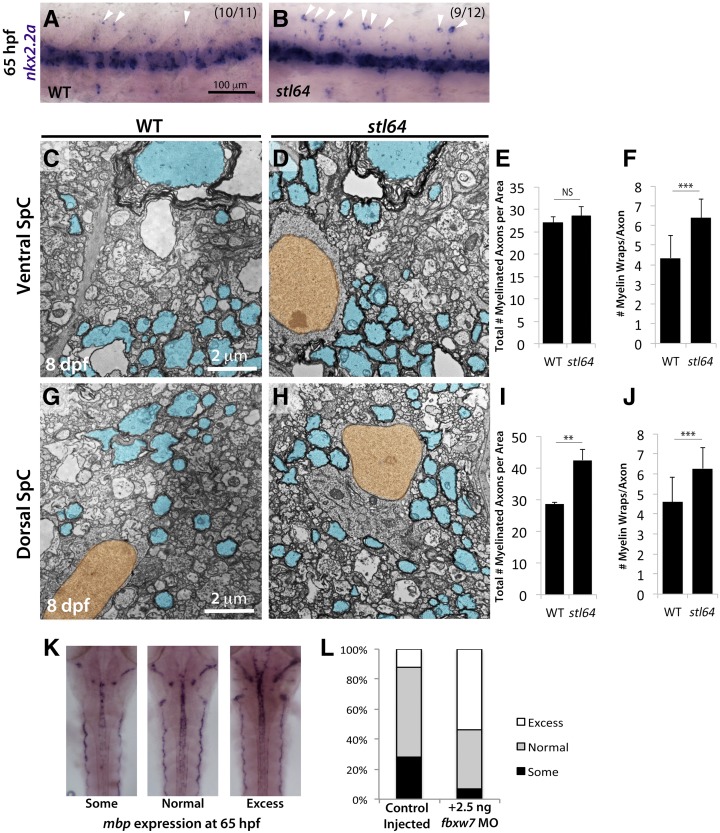
The *fbxw7^stl64^* allele phenocopies the *fbxw7^vu56^* allele. (A and B) *stl64* mutants display more *nkx2.2a*+ cells in the dorsal spinal cord (SpC) relative to their WT siblings at 65 hpf. TEM analysis of the ventral (C–F) and dorsal (G–J) SpC at 8 dpf shows that *fbxw7^stl64^* mutants have more myelinated axons in the dorsal SpC (I) and thicker myelin in both regions (F and J). (K and L) Transient morpholino (MO) knockdown of *fbxw7* in WT embryos results in *mbp* overexpression at 65 hpf compared to control-injected siblings. Error bars are SD. ** *P* < 0.01, *** *P* < 0.001. NS, not significant.

Previously, zebrafish *fbxw7^vu56^* mutants were shown to possess an increased number of oligodendrocytes and to display hyper-myelination in the CNS (Snyder *et al.* 2012; Kearns *et al.* 2015). To determine if the overexpression of *mbp* in the CNS of *stl64* mutants was similarly due to increased oligodendrocyte numbers, we performed WISH using a riboprobe against *nkx2.2a*, which marks the oligodendrocyte lineage. At 65 hpf, *stl64* mutants displayed more *nkx2.2a*-positive cells in the spinal cord than their WT siblings (Figure 4, A and B). Further, ultrastructural analyses by TEM revealed increased numbers of myelinated axons as well as thicker myelin in the spinal cords of *stl64* mutants at 8 dpf (Figure 4, C–J). To further test if *fbxw7* regulates myelination, we employed an established antisense oligonucleotide morpholino (MO) (Snyder *et al.* 2012) to reduce *fbxw7* levels in WT embryos. We then assessed *mbp* expression by WISH, and found that larvae injected with low doses of the *fbxw7*-MO displayed increased *mpb* expression (Figure 4, K and L). Together, our WGS mapping using fixed tissues, the phenotypic similarities of *stl64* and *fbxw7^vu56^* mutants, and our *fbxw7*-MO analyses strongly support that the *stl64* mutation represents a new allele of the *fbxw7* gene.

## Discussion

Here, we have described a WGS–BSA pipeline for identifying the causative mutations for phenotypes discovered in zebrafish forward genetic screening strategies requiring fixed tissue. We tested this protocol by mapping two alleles, *gpr126^st49^* and *egr2b^fh227^*, known to disrupt myelination. Furthermore, we successfully mapped a novel allele, *stl64*, generated in a WISH-based forward genetic screen, to a nonsense mutation in the gene *fbxw7*.

In validating the WGS–BSA pipeline, the importance of genetic variation in the lines sequenced became clear with the sequencing of the *gpr126^st49^* pools. There were three linkage peaks for the *gpr126^st49^* pools compared to only a single linkage peak in the *egr2b^fh227^* and *fbxw7^stl64^* pools. We believe this is due to the crossing history of each line. The *egr2b^fh227^* line was maintained on an AB* (ZFIN ID: ZDB-GENO-960809-7) background with intermittent outcrossing to the wild-caught, IND line (ZFIN ID: ZDB-GENO-980210-28). Similarly, the screen that generated the *fbxw7^stl64^* allele started on the SAT background (ZFIN ID: ZDB-GENO-100413-1) before being outcrossed to SJD (ZFIN ID: ZDB-GENO-990308-9) (Johnson *et al.* 1996) and other fish with mixed backgrounds (pigment mutants and transgenes) before sequencing. The outcrossing of both the *egr2b^fh227^* and *fbxw7^stl64^* lines drove down the level of homozygosity across their respective genomes and allowed the difference in ratio between the sibling and mutants pools to be more easily observed. In contrast, the *gpr126^st49^* line in our laboratory has been maintained exclusively on the AB* background without intermittent outcrossing. This difference in crossing history has driven up the level of homozygosity and led to three distinct regions of the genome being linked to the *st49* phenotype. This large linkage peak underscores the importance of performing at least one outcross before collecting mutants for pooled sequencing.

It was also apparent from our analyses that coverage of the genome when using WGS was especially important for filtering of putatively causative SNPs. For *st49*, in the middle of the third linked region on chromosome 20 (spanning 37.4–41.6 Mb), eight bins had <10 SNPs fall into them and were subsequently filtered out. This differs from both the *fh227* and *stl64* sequencing, which had hundreds of SNPs across the entire region. The loss of the eight bins in the middle of the region likely suppressed the expected signal from the true *gpr126^st49^* mutation, contributing to the generally broad linkage observed. Additionally, when verifying the homozygosity of the *gpr126^st49^* allele in the mutant sequencing, a single read was all that covered the mutation in the *st49* sibling pool, further underscoring the importance of coverage depth in calling variants. Based on the sequencing described here, a target of 100-million mapped reads is the minimal target recommended for future experiments.

We have shown that linking a genomic region to a phenotype using our WGS–BSA approach is relatively robust and is able to overcome excessive inbreeding and relatively low coverage; however, determining if a specific mutation from the linked region is causative has several important caveats. The filtering methods described here are specific to defining coding variants introduced by mutagenesis and are not applicable to all scenarios. For example, in the case of an allele that was simply an endogenous, rare, recessive mutation driven to homozygosity in the process of the screen, the SNPFisher database of naturally occurring variants would be inappropriate to use as a filter. Similarly, if the responsible mutation is in a regulatory region, limiting SNPs to only those designated as coding by snpEFF is similarly inappropriate. In both these cases, SNPs previously observed as homozygous should still be removed, but the SNPFisher database should not be included.

In the case where an allele’s causative mutation is a nonsynonymous mutation introduced through mutagenesis, as opposed to the three nonsense mutations described here, we propose a schema to prioritize the most likely causative mutation. Highest priority should be given to mutations affecting splice sites or out-of-frame INDELS. Below that, priority should be given to nonsynonymous SNPs that disrupt protein domains or cause shifts in polarity or hydrophobicity between amino acid side chains. The mutation with the highest probability to be causative should be confirmed by creation of a second allele and complementation testing.

Despite the caveats regarding coverage, outcrossing, and SNP filtering, our demonstrated ability to extract gDNA from fixed tissue in order to map and characterize mutations is important. Using WGS with BSA over traditional PCR-based mapping methods can dramatically shrink the time required to map some mutants. Including fixed tissue as a DNA source for this procedure opens up this approach for a cadre of mutants not easily identified by gross morphological changes. As a result, many new genes may be discovered from existing mutants, and new screens can be envisioned involving these types of screens using fixed zebrafish tissues. We note that our protocol to obtain high-quality gDNA from fixed tissue can be paired with any number of useful pipelines already described to define causative lesions by WGS (*e.g.*, Henke *et al.* 2013).

In conclusion, as WGS has increased the speed by which mutants discovered in forward genetic screens can be linked to a causative region and mapped to a specific lesion, the inability to use WGS to map alleles that need to be phenotyped using fixed tissue has held back the mapping of an entire class of alleles. To address this hurdle, we created a WGS–BSA pipeline for sequencing and analysis of gDNA extracted from pools of fixed or fresh tissue. This pipeline compares areas of the genome between mutant and sibling pools to calculate the ratios of variant to reference alleles for that specific region. Furthermore, filtering out natural variants and variants previously observed as homozygous from the list of predicted protein-coding variants in the linked regions allows for an accurate attribution of causality to a specific mutation. Using this WGS-based mapping pipeline, we correctly identified the previously described mutations, *gpr126^st49^* and *egr2b^fh227^*, when gDNA was extracted from pools of either fresh or fixed tissue, respectively. To test the ability of the WGS–BSA pipeline to map a novel mutant, we used this pipeline to identify the causative lesion in an unidentified CNS hyper-myelination mutant, designated *stl64*, from our WISH-based forward genetic screen. We found that *stl64* was most strongly linked to a 3-Mb region of chromosome 1, with the most deleterious mutation being a C-to-A transition leading to a PTC in the gene *fbxw7*. Importantly, *stl64* mutants phenocopy the CNS myelination defects observed in a previously described mutation in the same gene, *fbxw7^vu56^*. Finally, loss of *fbxw7* in WT zebrafish larvae resulted in similar overexpression of *mbp*, further supporting the conclusion that *stl64* disrupts the gene *fbxw7*. These experiments demonstrate that our new pipeline can successfully identify causative lesions for mutants that can only be analyzed using fixed tissue preparations. We believe that this pipeline can be easily applied to extract gDNA from pooled fixed or fresh tissue for WGS with BSA to determine the protein-coding causative mutation in zebrafish screen mutants.
